# Bacillus Subtilis 29784 as a Feed Additive for Broilers Shifts the Intestinal Microbial Composition and Supports the Production of Hypoxanthine and Nicotinic Acid

**DOI:** 10.3390/ani11051335

**Published:** 2021-05-08

**Authors:** Pearl Choi, Lamya Rhayat, Eric Pinloche, Estelle Devillard, Ellen De Paepe, Lynn Vanhaecke, Freddy Haesebrouck, Richard Ducatelle, Filip Van Immerseel, Evy Goossens

**Affiliations:** 1 Livestock Gut Health Team, Department of Pathology, Bacteriology and Avian Diseases, Ghent University, Salisburylaan 133, 9820 Merelbeke, Belgium; Pearl.Choi@Ugent.be (P.C.); richard.ducatelle@ugent.be (R.D.); 2 Adisseo France SAS, Center of Expertise and Research in Nutrition (CERN), 6 Route Noire, 03600 Commentry, France; Lamya.Rhayat@adisseo.com (L.R.); Eric.Pinloche@adisseo.com (E.P.); Estelle.Devillard@adisseo.com (E.D.); 3 Laboratory of Chemical Analysis, Ghent University, Salisburylaan 133, 9820 Merelbeke, Belgium; ellen.depaepe@ugent.be (E.D.P.); Lynn.Vanhaecke@ugent.be (L.V.); 4 Department of Pathology, Bacteriology and Avian Diseases, Ghent University, Salisburylaan 133, 9820 Merelbeke, Belgium; freddy.haesebrouck@ugent.be

**Keywords:** probiotics, *Bacillus subtilis*, metabolites, intestinal health, nicotinic acid, hypoxanthine, 16S rRNA gene sequencing, broilers

## Abstract

**Simple Summary:**

Bacterial strains that are consumed by humans or by animals to promote health are called probiotics. In poultry, *Bacillus* strains are widely used as feed additives for this purpose. Although different modes of action have been proposed, studies showing effects on what metabolites the bacteria produce in a test tube, and whether these can also be found in the intestine of animals that were given these strains as feed additives, are lacking. In the current study, we show that administration of a *Bacillus* strain to broiler chickens changes the microbial composition in the gut by reducing opportunistic pathogenic bacterial families and promoting beneficial bacterial families. We show that two molecules, hypoxanthine and nicotinic acid, are produced by the *Bacillus* strain and are elevated in the intestinal tract of these animals. We hypothesize that nicotinic acid can be used by beneficial microbes and is essential for their intestinal colonization, and that both molecules can have a positive effect on the intestinal wall. These data can be used to evaluate and develop novel feed additives to promote health of chickens, and reduce the need for antibiotic usage.

**Abstract:**

The probiotic *Bacillus subtilis* strain 29784 (Bs29784) has been shown to improve performance in broilers. In this study, we used a metabolomic and 16S rRNA gene sequencing approach to evaluate effects of Bs29874 in the broiler intestine. Nicotinic acid and hypoxanthine were key metabolites that were produced by the strain in vitro and were also found in vivo to be increased in small intestinal content of broilers fed Bs29784 as dietary additive. Both metabolites have well-described anti-inflammatory effects in the intestine. Furthermore, Bs29784 supplementation to the feed significantly altered the ileal microbiome of 13-day-old broilers, thereby increasing the abundance of genus *Bacillus*, while decreasing genera and OTUs belonging to the *Lactobacillaceae* and *Enterobacteriacae* families. Moreover, Bs29784 did not change the cecal microbial community structure, but specifically enriched members of the family *Clostridiales VadinBB60*, as well as the butyrate-producing families *Ruminococcaceae* and *Lachnospiraceae.* The abundance of various OTUs and genera belonging to these families was significantly associated with nicotinic acid levels in the cecum, suggesting a possible cross-feeding between *B. subtilis* strain 29784 and these beneficial microbes. Taken together, the data indicate that Bs29784 exerts its described probiotic effects through a combined action of its metabolites on both the host and its microbiome.

## 1. Introduction

Probiotics are used in both human and animal nutrition for their health benefits. In animal diets, probiotics are included as feed additives to create a healthy and resilient intestinal microbial environment [1,2,3]. Maintaining a beneficial intestinal microbial composition helps in improving the overall health of the animal and thereby positively affects body weight gain (BWG) and feed conversion ratio (FCR) [4,5].

Many different microorganisms are used as probiotics in poultry production. *Bacillus* spp. are the most commonly used probiotic microorganisms because of their ability to form endospores [6]. This enables them to survive the feed manufacturing process and the passage through the stomach. Moreover, spores allow easy administration, storage and prolonged shelf-life [6]. One frequently used species, *Bacillus subtilis*, is considered to be safe for consumption [7,8]. A variety of *B. subtilis* strains are available as feed additives for animals with each having their own strain specificity. One example is *B. subtilis* strain 29784 (Bs29784), for which beneficial effects on growth performance are consistently reported in broilers, turkeys and layer pullets [9,10,11,12]. In addition, the strain reduces IL-8 expression and improves intestinal barrier integrity by upregulating tight junction protein expression, as was shown in a cell culture model [13]. Although effects of the administration of *Bacillus* strains on intestinal health parameters have been observed, insights in the exact modes of action of these probiotic strains are often limited. Different modes of action have been suggested in literature, including vitamin and nutrient production, enzyme production, antagonistic effects on pathogens, pH reduction due to short-chain fatty acids (SCFA) and lactate production, amongst others, but causal relationships between the produced metabolites and the observed effects are generally not proven [14,15,16]. Studies investigating the metabolites produced by probiotic strains have focused mainly on fermentation products such as lactic acid and SCFA, while, to the best of our knowledge, none have carried out a metabolome analysis and verified whether the metabolites produced in vitro could also be detected in the intestinal tract. Therefore, the aim of the current study was to identify metabolites that are produced by the probiotic *B. subtilis* strain Bs29784 in vitro, elucidate whether these metabolites are also produced in the chicken intestinal tract after in-feed supplementation of Bs29784, and how Bs29784 affects the intestinal microbiome.

## 2. Materials and Methods

### 2.1. Bacterial Strain and Growth Conditions

Bs29784 is a commercially available probiotic for broilers (Alterion^®^ NE, Adisseo, Commentry, France). The commercial product contains 10^10^ CFU/g spores of *B. subtilis* strain 29784 and is mixed at 1 g/kg in the feed that is supplied to broilers. For in vitro experiments, a pure culture of Bs29784 was obtained by inoculating the commercial probiotic in Luria–Bertani (LB) broth (Sigma-Aldrich, St. Louis, MO, USA). The bacteria were grown overnight at 37 °C under aerobic conditions. Bacteria were plated on LB plates and their identity was confirmed via matrix-assisted laser desorption/ionization time of flight mass spectrometry (MALDI-TOF MS) [17] and Sanger sequencing of the 16S region [18]. Bacterial growth was determined in LB broth over a 24-hour time span (grown in triplicate). Bacterial supernatant was obtained by centrifugation (5 min, 13,300 rpm) and filtered using a Polyvinylidene difluoride (PVDF) membrane filter (0.22 µm × 13 mm diameter, Kynar 500^®^, Arkema, Amsterdam, The Netherlands). Blank samples (medium without bacteria) were incubated simultaneously with the bacterial samples and processed in the same way to serve as controls. Samples were stored at −80 °C until metabolomic analysis.

### 2.2. Animal Trial

The study was undertaken following the guidelines of the ethics committee of the Faculty of Veterinary Medicine, Ghent University, in accordance with the EU Directive 2010/63/EU. One-day-old Ross 308 broiler chicks were obtained from a local hatchery and divided into 2 groups of 5 birds consisting of (1) a control group that received a standard commercial diet and (2) a group that received a standard commercial diet supplemented with the commercial Bs29784 probiotic at a dose of 10^10^ CFU/kg feed (FARM 1&2 mash, Versele-laga, Deinze, Belgium). Animals were housed on a solid floor covered with wood shavings at a density of 5 birds/m^2^. Animals were subjected to a light schedule of 12 h light and 12 h dark. All broilers were given water and feed ad libitum. At 13 days of age, all birds were weighed, the birds were euthanized, and digestive content from the jejunum, ileum and cecum was collected. These samples were frozen in liquid nitrogen directly after sampling and stored at −20 °C until further processing. The material from the 3 sections was used for metabolomic analysis and *Bacillus* quantification, while the ileal and cecal content was used for 16S sequencing. At 13 days of age, no differences in bodyweight could be observed, with an average bodyweight of 273.4 g ± 19.54 g (mean ± SD) for the control group and 254.3 g ± 38.37 g for the Bs29784-supplemented group (*p* = 0.358).

### 2.3. Targeted Metabolomics

#### 2.3.1. Reagents and Chemicals

Analytical standards [19] were obtained from Sigma-Aldrich (St. Louis, MO, USA), ICN Biomedicals Inc. (Costa Mesa, CA, USA) or TLC Pharmchem (Vaughan, ON, Canada). Solvents were obtained from Fisher Scientific UK and VWR International (Merck, Darmstadt, Germany). All analytical standards, including nicotinic acid (Sigma-Aldrich) and hypoxanthine (Sigma-Aldrich), as well as the internal standard valine-d8 (ISTD) (Sigma-Aldrich), were stored at −20 °C in a primary stock solution of 10 mg/mL in either ultrapure water or methanol.

#### 2.3.2. Instrumentation

A polar metabolomics approach was applied based on the method described by Vanden Bussche et al. (2015) [20]. An Accela UHPLC system of Thermo Fisher Scientific (San José, CA, USA) was used, with an Acquity HSS T3 C18 column (1.8 μm, 150 mm × 2.1 mm, Waters). As binary solvent system, ultrapure water with 0.1% formic acid (A) and acetonitrile acidified with 0.1% formic acid (B) were used at a constant flow rate of 0.4 mL/min. A gradient profile of solvent A was applied (0−1.5 min at 98% (*v*/*v*), 1.5−7.0 min from 98% to 75% (*v*/*v*), 7.0−8.0 min from 75% to 40% (*v*/*v*), 8.0−12.0 min from 40% to 5% (*v*/*v*), 12.0−14.0 min at 5% (*v*/*v*), 14.0−14.1 min from 5% to 98% (*v*/*v*)), followed by 4.0 min of re-equilibration. Solvents used for UHPLC-MS/MS analysis were purchased from Fisher Scientific UK. HRMS analysis was performed on an Exactive stand-alone benchtop Orbitrap mass spectrometer (Thermo Fisher Scientific), equipped with a heated electrospray ionization source (HESI), operating in polarity switching mode.

#### 2.3.3. Optimization of the UHPLC-HRMS Method

Optimization of the method of Vanden Bussche et al. (2015) [20] was performed in a preliminary run to exclude matrix effects and to determine the optimal concentration of the bacterial supernatant samples. For this purpose, quality control (QC) samples made from pooled biological samples were considered as representative bulk control samples [21]. QC samples were extracted and serially diluted with ultrapure water (1; 1:2, 1:5, 1:10, 1:20, 1:50, 1:100, 1:200 and 1:500), after which the linearity was studied based on the coefficient of determination (R^2^). The targeted analysis was based on an in-house metabolite mixture containing 291 known metabolites which are important in the gut. This mixture of metabolites was run to standardize and determine respective peaks found in the samples [22]. The absolute peak areas of the ISTD and of one representative metabolite from each category (multicarbon acids, monosaccharide, amino acid, imidazole, ketones, etc.) in the list of known metabolites was determined. The following 11 metabolites were analyzed: inositol, phenylacetic acid, succinate, histidyl leucine, glucose, 2-octanon, L-methionine, L-arginine, spermidine, hypoxanthine and uracil. The validated metabolites were required to have an R^2^ > 0.990. After validation, it was decided that a 1/10 dilution was optimal for the supernatant samples.

#### 2.3.4. Metabolomic Analysis

Metabolites produced by Bs29784 in vitro were analyzed together with blank samples. In vivo metabolite production was determined using intestinal digesta from chickens receiving either non-supplemented feed or feed supplemented with Bs29784. Therefore, intestinal content of the jejunum, ileum or cecum was freeze-dried for 24 h. To 100 mg of freeze-dried material, 2 mL of ice-cold methanol (80:20) was added, vortexed and centrifuged (9000 rpm, 10 min), after which the supernatant was filtered using a PVDF filter (0.45 µm × 25 mm diameter) and used at a 1:3 dilution. Xcalibur 3.0 software (Thermo Fisher Scientific, San José, CA, USA) was employed for targeted data processing, whereby compounds were identified based on their *m*/*z*-value, C-isotope profile, and retention time relative to that of the internal standard.

### 2.4. DNA Extraction from Intestinal Content

DNA was extracted from the jejunal, ileal and cecal content, using the hexadecyltrimethylammonium bromide (CTAB) method described by Griffiths et al. [23] with modifications described by Aguirre et al. [24]. The resulting DNA was resuspended in 50 µL of a 10 mM Tris-HCl buffer (pH 8.0) and the quality and concentration of the DNA was examined spectrophotometrically (NanoDrop, Thermo Fisher Scientific, Merelbeke, Belgium).

### 2.5. Quantification of Bacillus spp. and Total Bacteria

The percentage bacteria belonging to the genus Bacillus (*Bacillus* spp.) relative to the total number of bacteria found in the content from different intestinal segments was determined using quantitative PCR (qPCR). Primers targeting *Bacillus* spp. (YB-P1 and YB-P2) were used as described by Han et al. (2012) [25]. To determine the number of total bacteria, primers Uni 331F and Uni 797R were used as described by Hopkins et al. (2005) [26]. The qPCR was performed using the SensiFAST™ SYBR^®^ No-ROX Kit (Bioline, London, UK) with a 0.5 µM primer concentration. The PCR amplification consists of DNA pre-denaturation at 95 °C for 2 min followed by 30 cycles of denaturation (95 °C for 15 s), annealing (60 °C for 30 s) and extension (72 °C for 50 s).

### 2.6. 16S rRNA Gene Amplicon Sequencing

The V3–V4 hypervariable region of the 16s rRNA gene was amplified by using the gene-specific primers S-D-Bact-0341-b-S-17 and S-D-Bact-0785-a-A-21 [27]. The PCR amplifications were performed as described by Aguirre et al. (2019) [24]. CleanNGS beads (CleanNA, Gouda, The Netherlands) were used to purify PCR products. The DNA concentration of the final barcoded libraries was measured with a Quantus fluorimeter and Quantifluor dsDNA system (Promega, Madison, WI, USA). The libraries were combined to an equimolar 5 nM pool and sequenced with 30% PhiX spike-in using the Illumina MiSeq v3 technology (2 × 300 bp, paired-end) at the Oklahoma Medical Research center (Oklahoma City, OK, USA).

Demultiplexing of the amplicon dataset and deletion of the barcodes was done by the sequencing provider. Quality of the raw sequence data was evaluated using the FastQC quality control tool (Babraham Bioinformatics, Cambridge, UK), followed by an initial quality filtering with Trimmomatic v0.38 [28]. Reads with an average quality per base below 15 were cut using a four-base sliding window and reads with a minimum length below 200 bp were discarded. The paired-end sequences were assembled and primers were removed using PANDAseq [29], with a quality threshold of 0.9 and length cut-off values for the merged sequences between 390 and 430 bp. Chimeric sequences were removed using UCHIME [30]. Open-reference operational taxonomic unit (OTU) picking was performed at 97% sequence similarity using USEARCH (v6.1) and converted to an OTU table [31]. OTU taxonomy was assigned against the Silva database (v132, clustered at 97% identity) [32] using the PyNast algorithm with QIIME (v1.9.1) default parameters [33]. OTUs with a total abundance below 0.01% of the total sequences were discarded [34]. Potential contaminant chloroplastic and mitochondrial OTUs were removed from the dataset, resulting in an average of approximately 76,080 reads per sample, with a minimum of 25,725. Alpha rarefaction curves were generated using the QIIME “alpha_rarefaction.py” script and a subsampling depth of 25,000 reads was selected.

### 2.7. Metabolic Function Prediction of the Microbial Communities

Functional genes (KEGG orthologues, KOs) were inferred from the 16S amplicon sequencing data using Phylogenetic Investigation of Communities by Reconstruction of Unobserved States (PICRUSt), as previously described [24,35]. The resulting KEGG orthologues were further summarized into functional modules based on the Gut-specific Metabolic Modules (GMM) database using GoMixer (Raes Lab) [36,37]. The contribution of various taxa to different GMMs was computed with the script “metagenome_contributions.py”.

### 2.8. Statistical Analyses

Statistical analyses of the metabolomic and qPCR data were performed using GraphPad PRISM (v8.4.3). A Kolmogorov–Smirnov test was performed to evaluate the data for normal distribution. In case of normal distribution, an independent samples t-test was performed. When data were not normally distributed, a non-parametric Mann–Whitney test was performed. Tests were considered statistically significant at a *p*-value ≤0.05. Biologically relevant metabolite production by Bs29784 in vitro was identified as a fold change >2 and *p* < 0.05.

Statistical analyses of the 16S data were performed using R (v3.6.0). Alpha diversity was measured based on the observed OTUs (or observed KOs for the functional data), Chao1 and Shannon diversity index using the *phyloseq* pipeline [38]. Differences in alpha diversity were assessed using a Wilcoxon’s rank sum test. Beta diversity was calculated using Bray–Curtis distance. Differences in beta diversity were examined by permutational analysis of variance (Permanova) using the *adonis* function from the *vegan* package [39]. Differences in relative abundance at the phylum and family level were assessed using the two-sided Welch t-test from the *mt* wrapper in *phyloseq*, with the *p*-value adjusted for multiple hypothesis testing using the Benjamini–Hochberg method. The DESeq2 algorithm was applied to identify differentially abundant genera or functional modules between the control and Bs29784 group [40]. Significant differences were obtained using a Wald test followed by a Benjamini–Hochberg multiple hypothesis correction. For all tests, an adjusted *p*-value (*q*-value) ≤0.05 was considered significant. Biologically relevant differences in functional modules between the birds fed a control diet or Bs29784-supplemented diet were selected using a Log2 fold change (Log2FC) > 2 and *q*-value < 0.1.

The association of microbial abundances (at family, genus or OTU level), with hypoxanthine and nicotinic acid levels measured in the intestinal content, were analyzed using the multivariate analysis by linear models (MaAsLin2) R package. MaAsLin2 analysis was performed separately on the ileal and cecal samples, while controlling for treatment covariates [41].

## 3. Results

### 3.1. Identification of Metabolites Produced by Bs29784 In Vitro

Metabolites produced by Bs29784 after 24 h growth in LB medium were compared to the blank medium. Overall, 123 of the 291 targeted metabolites could be detected in either the blank LB medium and/or the supernatants of Bs29784 grown in LB (Table S1). The majority of the detected metabolites (96/123, 78%) were not significantly altered after growth of Bs29784 in the LB medium. In total, 21 metabolites (17% of the detected metabolites) were significantly reduced due to growth of Bs29784 and 16 metabolites (13% of the detected metabolites) were produced by Bs29784 in vitro (Table S1). Biologically relevant metabolites were identified based on a fold change >2 and *p* < 0.05 (Table 1). The most discriminatory metabolites, nicotinic acid and hypoxanthine (*p* < 0.0001), were selected for evaluation in the in vivo samples.

### 3.2. Effect of Supplementation of Bs29784 in Broiler Feed on the Bacillus Load, Levels of Hypoxanthine and Nicotinic Acid in the Intestinal Tract

**Figure 1:**
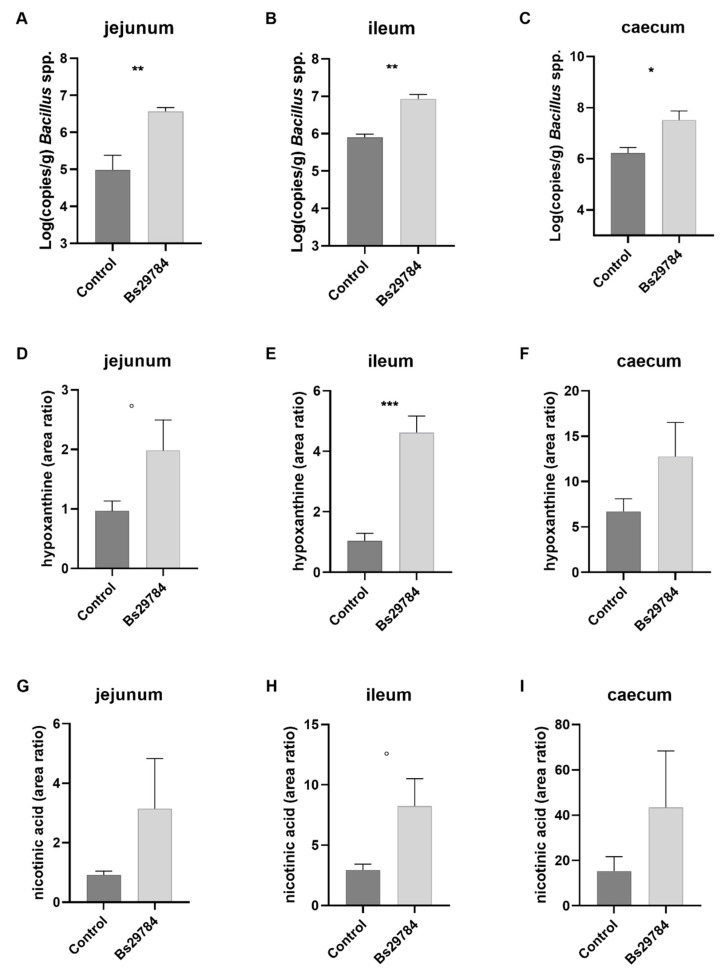
Abundance of *Bacillus* spp. and metabolite concentrations in jejunum, ileum and cecum. The *Bacillus* load in the jejunum, ileum and cecum was measured via qPCR (**A**–**C**). The metabolites hypoxanthine (**D**–**F**) and nicotinic acid (**G**–**I**) are expressed as area ratio. ° *p* < 0.1, * *p* < 0.05, ** *p* < 0.01, *** *p* < 0.001.

The total number of bacteria, as well as the number of *Bacillus* spp. in the jejunum, ileum and cecum were determined using qPCR. Supplementation of the diet with the probiotic *B. subtilis* strain Bs29784 did not introduce alterations in the total bacterial load (data not shown), but significantly increased the number of *Bacillus* spp. in the ileum (*p* = 0.005), jejunum (*p* = 0.008), and cecum (*p* = 0.014) (Figure 1A–C).

To further assess whether this increase in *Bacillus* spp. was reflected in an increase in Bs29784 metabolites, the levels of hypoxanthine and nicotinic acid were determined. Overall, broilers fed a Bs29784-containing diet showed higher levels of hypoxanthine and nicotinic acid in the intestinal content. The increase in hypoxanthine was most pronounced in the ileum (*p* = 0.0003), but did not reach significance in the jejunum (*p* = 0.095) or cecum (*p* = 0.171) (Figure 1D–F). In-feed supplementation of Bs29784 tended to increase the level of nicotinic acid in the ileum (*p* = 0.051), as compared to birds fed the control diet, but had no effect on nicotinic acid levels in the jejunum (*p* = 0.223) or cecum (*p* = 0.306) (Figure 1G–I).

### 3.3. Effect of Bs29784 Supplementation in Broiler Feed on the Ileal and Cecal Microbial Diversity

The microbial complexity in the ileum and cecum was estimated by calculating the number of observed OTUs, the estimated OTU richness (Chao1) or the estimated community diversity (Shannon index) in each sample. In-feed supplementation of Bs29784 had no effect on the ileal microbial richness (observed OTUs or Chao1) (Table 2). However, addition of Bs2978 to the diet significantly reduced the ileal community diversity (Shannon index, *p* = 0.032). This is in contrast to the situation in the cecum, which had a tendency for higher microbial richness in birds fed the Bs29784-supplemented diet, as compared to the control diet (observed OTUs: *p* = 0.056, Chao1: *p* = 0.15), but no effect of Bs29784 on the microbial community diversity was observed (Table 2).

**Figure 2:**
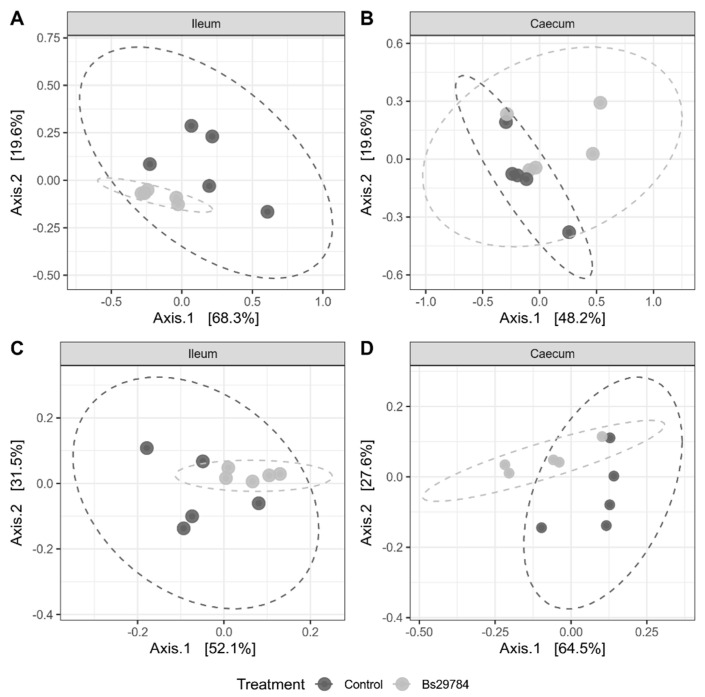
PCoA plot of the taxonomic and functional microbial diversity from birds fed a control or Bs29784-supplemented diet. Principal coordinate analysis (PCoA) plots of bacterial taxonomic (OTU-level) (**A**,**B**) or functional (KO-level) (**C**,**D**) diversity calculated using the Bray–Curtis dissimilarity metric. Each dot represents an individual chicken microbiome. Significant separation of the microbial communities was observed in the ileum (*p* = 0.028) (**A**), but not the cecum (*p* = 0.153) (**B**). In both the ileum and cecum, significant grouping of the samples was observed based on the functional KO diversity (*p* = 0.024 and *p* = 0.029) (**C**,**D**).

Bray–Curtis dissimilarity was used to investigate beta diversity between either the ileal or cecal microbiota from birds fed the control diet or the diet supplemented with *B. subtilis* strain 29874. Supplementation of Bs29784 to the broiler diet showed a significant clustering in the ileum, with 33.7% of the variation between the samples being explained by the Bs29784 supplementation to the feed (*p* = 0.028) (Figure 2A). However, no effect on the cecal microbial community composition was observed (diet explaining 17.4% of the variation, *p* = 0.15) (Figure 2B).

### 3.4. Influence of Bs29784 on the Taxonomic Composition of the Ileal and Cecal Microbiome

The most abundant phyla in the ileum were *Firmicutes* (84.94% in control, 96.83% in Bs29784) and *Proteobacteria* (12.81% in control, 2.24% in Bs29784), with a minor portion belonging to the *Verrucomicrobia* (1.97% in control, 0.80% in Bs29784) and *Actinobacteria* (0.28% in control, 0.13% in Bs29784). Also in the cecum, the *Firmicutes* was the most prevalent phylum in both groups (48.16% in control, 68.37% in Bs29784), followed by the *Proteobacteria* (26.27% in control, 10.54% in Bs29784) and *Verrucomicrobia* (24.29% in control, 19.68% in Bs29784). The phylum *Actinobacteria* accounted for 1.28% and 1.41% of the cecal microbiome in birds fed the control or Bs29784-supplemented diet, respectively. Addition of Bs29784 to the broiler diet had no significant influence on either the ileal or cecal microbiome at phylum level.

In the ileum, the families *Bacillaceae* (<0.001% in control, 0.12% in Bs29784, *p*_adj_ = 0.06) and *Enterococcaceae* (45.25% in control, 82.47% in Bs29784, *p*_adj_ = 0.17) tended to be more abundant after probiotic supplementation, whereas both the family *Leuconostocaceae* (0.25% in control versus 0.0016% in Bs29784, *p*_adj_ = 0.06) and family *Lactobacillaceae* (24.45% in control and 2.51% in Bs29784, *p*_adj_ = 0.17) tended to be less abundant in the ileum of birds fed the Bs29784-supplemented diet. No significant effect of Bs29784 supplementation on the families in the cecum could be observed.

**Figure 3:**
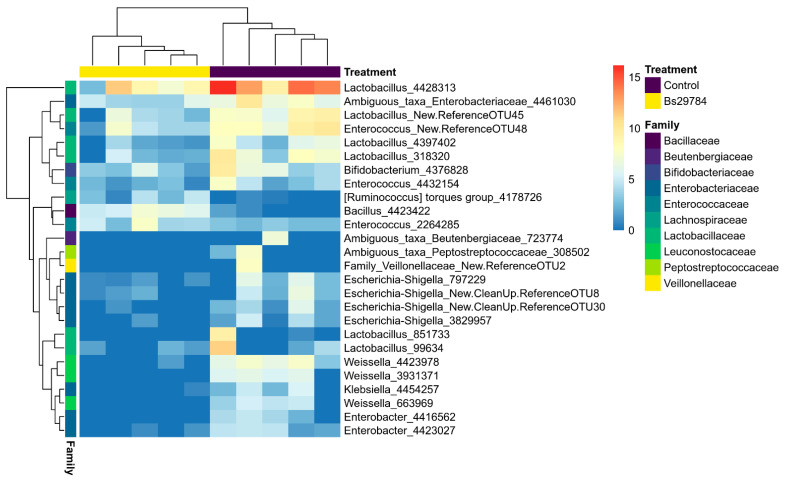
Differentially abundant OTUs in the ileal microbiome of birds fed either a control or Bs29784-supplemented diet. The abundance of the OTUs is shown as the log2 of the DESeq2-normalized counts. Each OTU is labelled with the genus information, or family information when no genus identification was possible, followed by the OTU number.

**Figure 4:**
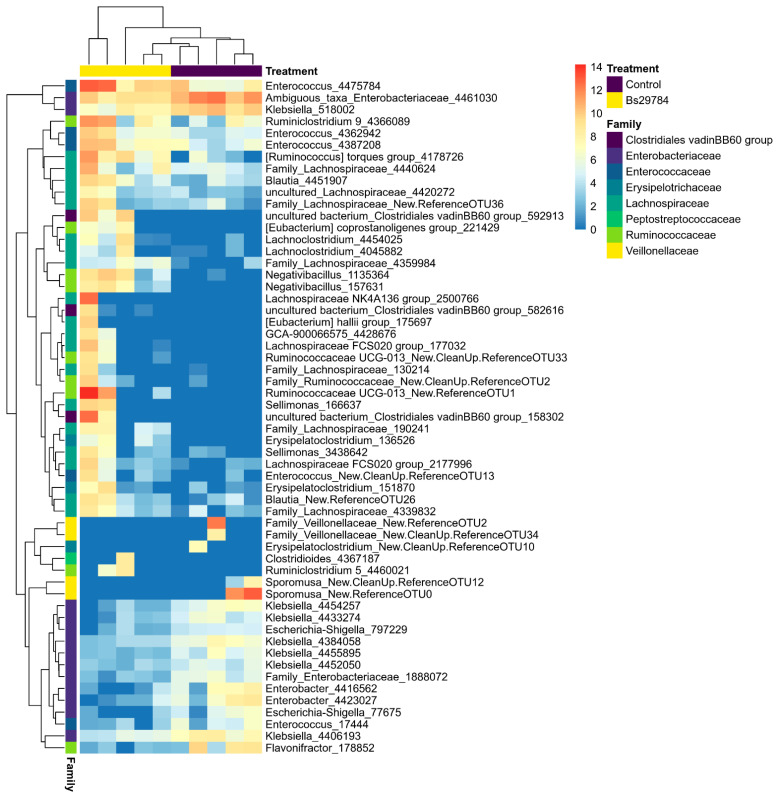
Differentially abundant OTUs in the cecal microbiome of birds fed either a control or Bs29784-supplemented diet. The abundance of the OTUs is shown as the log2 of the DESeq2-normalized counts. Each OTU is labelled with the genus information, or family information when no genus identification was possible, followed by the OTU number.

Differentially abundant genera and OTUs in the ileal or cecal microbiome from birds fed a Bs29784-supplemented diet as compared to the control diet were identified using DESeq2 (Table 3; Tables S2 and S3). Nine genera were differentially abundant between the ileal microbiota from birds fed either the control diet or the Bs29784 diet. Only the genus *Bacillus* was significantly increased in the ileal microbiota of birds fed the Bs29784-containing diet, a difference that could be fully attributed to a single OTU identified as *Bacillus subtilis* (OTU4423422, Figure 3, Table S2). The other significantly altered genera and OTUs in the ileal microbiome were all less abundant in Bs29784-fed birds, with multiple genera belonging to the *Enterobacteriaceae* family, including multiple OTUs belonging to genera *Escherichia-Shigella* and *Enterobacter* (Figure 3). Furthermore, addition of Bs29784 to the broiler feed resulted in a reduction of the genus *Pediococcus* and *Weissella*, as well as multiple OTUs belonging to the genus *Lactobacillus* in the ileal microbiome (Table 3, Figure 3). In the cecum, Bs29784 supplementation of the broiler feed significantly reduced the relative abundance of multiple genera belonging to the families *Veillonellacaea* and *Enterobacteriaceae,* with main OTUs belonging to the genus *Klebsiella* (Figure 4, Table S3). Additionally, an increase in members of the butyrate-producing families *Ruminococcaceae* and *Lachnospiraceae* was observed in the cecum of Bs29784-fed birds. Moreover, the genus *Enterococcus*, *Clostridioides* and a genus belonging to the *Clostridiales vadin*BB60 group were significantly increased in the cecum by Bs29784 supplementation of the feed (Table 3).

### 3.5. Hypoxanthine and Nicotinic Acid Levels Are Associated with Specific Microbial Taxa in the Cecum

**Figure 5:**
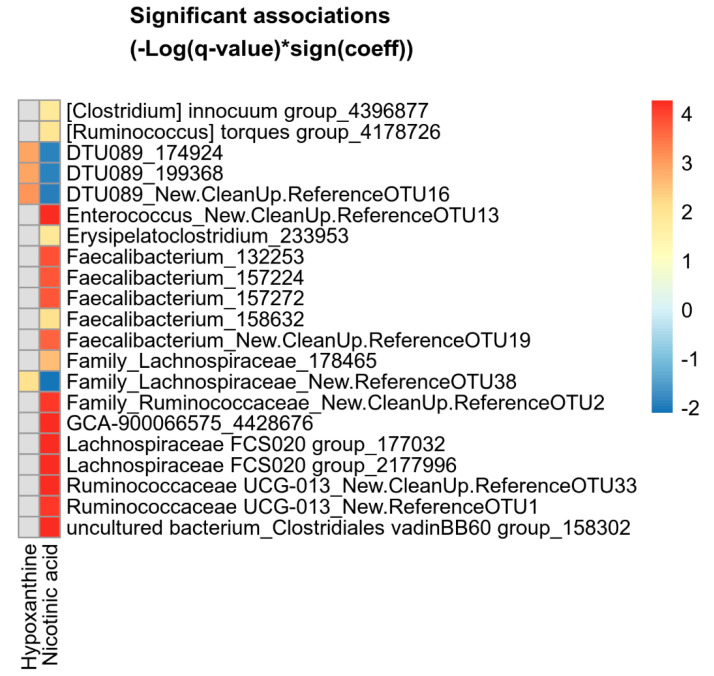
Heatmap of microbial OTUs showing significant association with hypoxanthine or nicotinic acid levels in the cecum. Significant associations were identified using MaAsLin2 and are plotted as (−Log(*q*-value)*sign(coeff.)). Grey squares: no significant association.

Associations between the hypoxanthine and nicotinic acid levels and microbial abundances in either the ileum or cecum were analyzed using multivariate association with linear models (MaAsLin2), while controlling for the type of diet (control diet or Bs29784-supplemented diet). In the ileum, no associations between metabolite levels and the abundance of specific microbial taxa were observed. In the cecum, the genus DTU089 (family *Ruminoccocaceae*) was significantly associated with the hypoxanthine levels (*p* = 0.001, *q* = 0.022) and inversely correlated with the nicotinic acid levels (*p* = 0.006, *q* = 0.099). These associations were also significant at the OTU level (Figure 5). Additionally, a similar association between metabolite levels and a single OTU belonging to the family *Lachnospiraceae* was observed (Figure 5). No other associations with hypoxanthine levels in the cecum could be observed. In contrast with the limited number of microbiome–hypoxanthine associations, the effect of nicotinic acid on the cecal microbiome was more pronounced. Nicotinic acid levels were positively associated with 17 OTUs, mainly ones belonging to the families *Lachnospiraceae* and *Ruminococcaceae* (Figure 5). Five out of seventeen OTUs (29.4%) that were associated with the cecal nicotinic acid levels belong to *Faecalibacteria*, and were mainly identified as *F. prausnitzii* (4/5 *Faecalibacterium* OTUs). These microbiome–nicotinic acid associations were also significant at the genus level, and even the family level, for both the family *Ruminococcaceae* (*p* = 0.012, *q* = 0.222) and family *Clostridiales vadinBB60 group* (*p* = 0.001, *q* = 0.024).

### 3.6. In-Feed Bs29784 Supplementation Decreases the Abundance of Specific Microbial Metabolic Modules

To determine whether the Bs29784-induced alterations of the microbiota might have an effect on the microbial functions, the functional genes (KEGG orthologs) present in the ileal and cecal microbiome were in silico predicted and grouped into gut-specific metabolic modules (GMMs). In total, 5135 and 4674 KOs were identified in, respectively, the ileal and cecal microbiome. In-feed supplementation of Bs29784 had no effect on both the ileal and cecal functional richness (number of observed KOs or Chao1 richness estimator), but reduced the diversity of the functional genes (Shannon diversity, ileum: *p* = 0.15, cecum: *p* = 0.016) (Table 2). Beta-diversity analysis based on Bray–Curtis showed significant clustering in both the ileum and cecum with 28.0% and 33.8% of the variation between the samples being explained by the Bs29784 supplementation to the feed (ileum: *p* = 0.024, cecum: *p* = 0.029) (Figure 2C,D).

Based on the identified functional genes, 127 and 126 gut metabolic functional modules (GMM) could be constructed in, respectively, the ileum and cecum. None of the GMMs were significantly more abundant in either the ileum or cecum from birds receiving the Bs29784-supplemented feed. However, 13 GMMs were significantly less abundant in the ileum, whereas 7 GMMs were reduced in the cecum of Bs29784-fed birds (Tables S4 and S5). The affected GMMs can be classified in seven functional categories: amines and polyamines degradation (MF004), amino acid degradation (MF0015, MF0024, MF0036, MF0037 and MF0041), carbohydrate degradation (MF0045, MF0052), gas metabolism (MF0095), inorganic nutrient metabolism (MF0104), lipid degradation (MF0106, MF0111) and organic acid metabolism (MF0118, MF0120, MF0125, MF0128).

**Figure 6:**
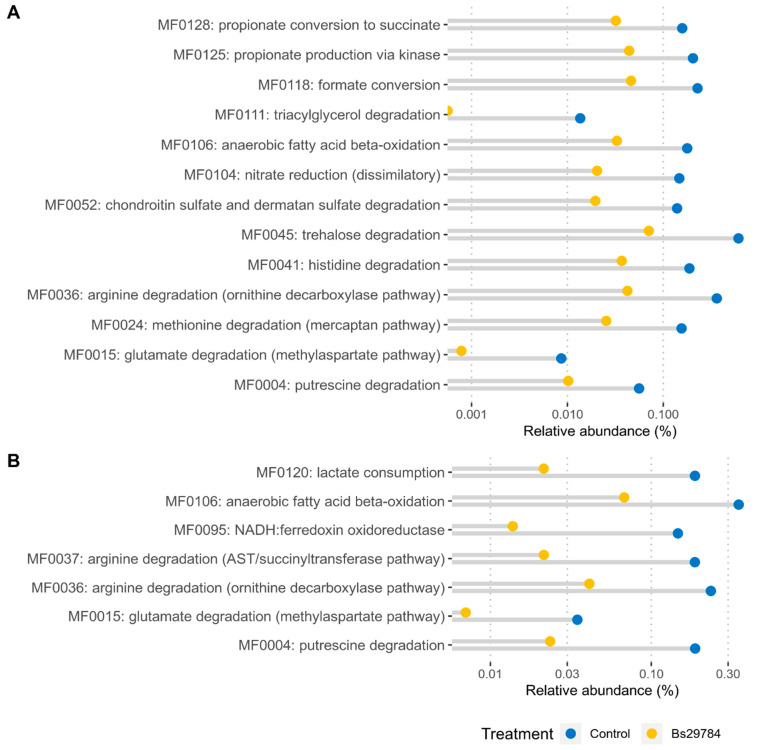
Relative abundances of gut-specific metabolic modules (GMMs) in ileum (**A**) or cecum (**B**) of broilers with control and Bs29784-supplemented diets. Functional modules with a Log2FC > 2 and *q*-values < 0.1 are shown.

**Figure 7:**
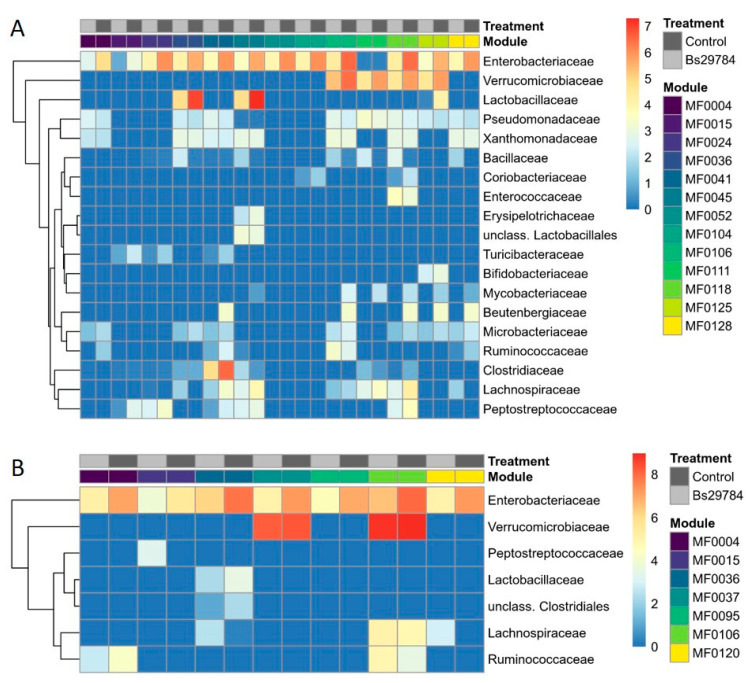
Bacterial families responsible for each of the functional modules detected in ileum (**A**) or cecum (**B**) of control animals and broilers supplemented with Bs29784 in the feed. Metagenome contributions on the family level are sorted per functional module and per treatment (control: dark gray; Bs29784: light gray). The log2 of the module counts per family are shown on a blue–red scale. Only families that were present in at least 3 out of 5 samples from either treatment group were included. MF0004: putrescine degradation, MF0015: glutamate degradation, MF0024: methionine degradation, MF0036: arginine degradation (ornithine decarboxylase pathway), MF0037: arginine degradation (AST/succinyltransferase pathway), MF0041: histidine degradation, MF0045: trehalose degradation, MF0052: chondroitin sulfate and dermatan sulfate degradation, MF0095: NADH:ferredoxin oxidoreductase, MF00104: nitrate reduction, MF0106: anaerobic fatty acid beta-oxidataion, MF0111: triacylglycerol degradation, MF0118: formate conversion, MF0120: lactate consumption, MF0125: propionate production via kinase, MF0128: propionate conversion to succinate.

To further address the metagenomic potential of the ileal and cecal microbiota, the relative abundance of the GMMs of interest (Figure 6) as well as the microbial taxa putatively contributing to the selected pathways were identified (Figure 7, Tables S6 and S7). In the ileum, the majority of the changes in predicted metabolic modules could, at least partly, be attributed to members of the family *Enterobacteriaceae* (Figure 7A). Additionally, the genus *Akkermansia* within the family *Verrucomicrobiaceae* contributed for a large part to the observed reduction of a selection of GMMs (MF0106, MF0111, MF0118, MF0125), which are mainly involved in lipid degradation and organic acid metabolism (Figure 7A). In addition to the family *Enterobacteriaceae*, the *Lactobacillaceae* were main contributors to the arginine degradation (MF0036) and trehalose degradation (MF0045) modules, whereas the *Clostridiaceae* were in large part responsible for the histidine degradation (MF0041) module. Other bacterial families had only minor taxonomic contributions to the differences in metabolic modules encoded by the ileal microbiome from broilers fed a control or Bs29784-supplemented diet (Figure 7A, Table S6).

In the cecum, members of the family *Enterobacteriaceae* were contributing greatly to the observed differences in metabolic modules (Figure 7B). This effect of the *Enterobacteriaceae* is partially counteracted by a taxonomic increase of the families *Ruminococcaceae* and *Lachnospiraceae*, which specifically contribute to the modules encoding for arginine degradation (MF0036), anaerobic fatty acid beta-oxidation (MF0106) and lactate consumption (MF0120) (Figure 7B). Additionally, the genus *Akkermansia* (family *Verrucomicrobiaceae*) had a large share in the abundance of modules MF0106 and MF0037, but it did not influence the overall module abundance (Figure 7B).

## 4. Discussion

The *Bacillus subtilis* strain 29784 was previously shown to improve growth performance in broilers, turkeys and layer pullets [10,11,12], have a beneficial effect on the gut mucosal morphology in broilers [9] and increase the abundances of butyrate-producing bacteria in the ceca of both broilers and layer pullets [9,42]. Moreover, Bs29784 was shown to possess anti-inflammatory properties and enhance epithelial barrier integrity in vitro [13]. However, how Bs29784 modulates the microbiome and interacts with the host was largely unknown. In this study, we identified nicotinic acid and hypoxanthine as important metabolites that might contribute to the above-described host- and microbiome-modulating effects of Bs29784. Indeed, nicotinic acid and hypoxanthine were produced by Bs29784 in vitro and were also increased in the ileum of broilers fed a Bs29784-supplemented diet. *Bacillus subtilis* spores have been found to germinate in the small intestine of mice [43] and chickens [44]. The observed increase of hypoxanthine and nicotinic acid in the small intestine of broilers fed a Bs29784-supplemented diet indicates that the Bs29784 spores were germinating in the intestine and suggests that *Bacillus*-produced metabolites are able to actively contribute to the metabolite pool produced by the gastrointestinal microbiome.

In-feed supplementation of Bs29784 induces a shift in the cecal microbiome towards butyrate-producing bacteria, which can at least partly be explained by the metabolites produced by Bs29784. Although no changes were observed in the overall community structure, Bs29784 specifically decreased the abundance of multiple genera belonging to the families *Veillonellaceae* and *Enterobacteriaceae*, while increasing members of the families *Clostridiales VadinBB60*, *Ruminococcaceae* and *Lachnospiraceae.* This is in accordance with previous studies in both broilers and layers, where *B. subtilis* strain 29784 increased the cecal abundance of the butyrate-producing families *Ruminococcaceae* and *Lachnospiraceae* [9,42]. In this study, we showed that the abundance of various OTUs and genera belonging to the *Clostridiales VadinBB60*, *Ruminococcaceae* and *Lachnospiraceae* was significantly associated with nicotinic acid levels in the cecum. A similar association between nicotinic acid levels in the gut and the genus *Faecalibacterium* was previously observed in samples from inflammatory bowel disease (IBD) patients [45]. In both IBD patients and in our study, this association could mainly be attributed to *Faecalibacterium prausnitzii*. As *F. prausnitzii* is auxotroph for nicotinic acid, it has to acquire this nutrient form the environment [46,47], suggesting possible cross-feeding between *B. subtilis* strain 29784 and *F. prausnitzii* in the gut. Moreover, various members of the *Ruminococcaceae* and *Lachnospiraceae* lack the pathways for de novo synthesis of several other B-vitamins (mostly vitamin B1 (thiamin), B5 (pantothenate), B6 (pyridoxine) and B7 (biotin)), while these pathways were encoded in the genome of various *B. subtilis* strains [48]. Therefore, it might be that the observed association between nicotinic acid and these bacteria is caused by the production of other B vitamins by Bs29784. Indeed, we showed that Bs29784 is able to produce pantothenate in vitro. However, this vitamin was not further investigated in this study. Whether or not Bs29784 is able to produce other B-vitamins and steer the microbiome towards an anti-inflammatory community through cross-feeding remains to be elucidated.

Bs29784 addition to the broiler diet changes the microbial community structure in the ileum, thereby mainly reducing the abundance of various genera and OTUs belonging to the *Lactobacillaceae* and *Enterobacteriaceae*, while increasing the abundance of *B. subtilis*. This is in contrast to a previous study where in-feed supplementation of Bs29784 had no effect on the ileal microbiome in broilers [9]. This difference might be attributed to the age of the birds, where the aforementioned study used 42-day-old broilers, while our study aimed at studying the more dynamic microbiome of 13-day-old birds. Moreover, supplementation of *B. subtilis* strain 29784 in the feed of broilers reduced the abundance of several functional modules, which were mainly involved in amino acid degradation or organic acid metabolism. This effect on the microbial functional potential was less pronounced in the cecal microbiome and was in large part due to a reduction in *Enterobacteriaceae.* As no association was observed between hypoxanthine or nicotinic acid levels and the microbiome in the ileum, it is unclear how Bs29784 exerts its microbiome-modulating effect in the ileum. One possibility is that the observed microbiome effects are caused by the production of anti-microbial peptides by Bs29784 or through an indirect effect of Bs29784 on the host. Alternatively, it might be that the number of animals used in this study (*n* = 5 per group) did not yield enough statistical power to discover possible associations between the Bs29784-produced metabolites and the ileal microbiome.

In addition to the abovementioned effects on the microbiome, beneficial effects on intestinal health for both hypoxanthine and nicotinic acid were previously reported. Reduced faecal levels of hypoxanthine or nicotinic acid have both been linked with IBD [45,49,50]. Furthermore, both metabolites are able to ameliorate experimental colitis [51,52]. Additionally, nicotinic acid treatment promoted mucosal healing in patients with moderately active ulcerative colitis [51].

Hypoxanthine is a breakdown product of nucleic acids and can be taken up and incorporated by intestinal bacteria or the host via the nucleotide salvage pathway [53]. Additionally, hypoxanthine from the microbiota is salvaged for energy and nucleotide biosynthesis in intestinal epithelial cells, thereby supporting wound healing, mucus generation and intestinal barrier function [49,52,54]. Notably, hypoxanthine has also been shown to act as a substrate for the antimicrobial function of the enzyme xanthine oxidoreductase (XOR) which is located on the outer surface of epithelial cells [38,39]. XOR is responsible for the conversion of hypoxanthine to xanthine and from xanthine to uric acid. During both reactions, oxygen is reduced, generating hydrogen peroxide (H_2_O_2_) and reactive oxygen species (ROS) [55,56]. XOR-generated H_2_O_2_ has been shown to act as an effective antimicrobial agent against commensal microorganisms and anaerobes, although pathogenic bacteria could be more resistant [56]. Moreover, XOR-generated ROS have been hypothesized to initiate neutrophil infiltration in response to pro-inflammatory mediators [57]. These neutrophils can then help to combat infections. In chickens, XOR is mainly expressed in the intestine, liver and pancreas [58]. It is thus possible that hypoxanthine produced by Bs29784 contributes to intestinal health through enhancing epithelial barrier function and mucus production, while protecting the intestinal epithelial cells against microorganisms through H_2_O_2_ production. This could be one of the reasons a reduction in several genera of the Enterobacteriaceae, such as *Enterobacter* and *Escherichia-Shigella*, is seen in the ileum of broilers fed Bs29784-supplemented feed.

Nicotinic acid, or niacin (pyridine-3-carboxylic acid), is a form of vitamin B3, an essential nutrient for animals, including broilers. In humans and rodents, nicotinic acid is known to bind on the GPR109A receptor (aka HCA2 or HM74a in humans and NIACR1 in rodents), which is also one of the receptors for butyrate [59,60,61]. GPR109A has been shown to act as an anti-inflammatory mediator via the β-arrestin signaling pathway, protecting epithelial cells against inflammation and oxidative stress [61]. It is unclear whether nicotinic acid induces similar effects in birds, since an equivalent homologous receptor has not yet been identified. Nevertheless, nicotinic acid shows comparable effects on the regulation of the lipid transport apolipoproteins apoA and apoB in broilers as in humans which is mediated by GPR109A in the latter [62]. Furthermore, nicotinic acid is an important precursor for the coenzymes nicotinamide adenine dinucleotide (NAD) and nicotinamide adenine dinucleotide phosphate (NADP) that play an essential role in, among others, antioxidant protection [63,64]. This suggests that nicotinic acid, produced among others by Bs29784, may be taken up by the epithelial cells, protecting the cells from oxidative stress, while at the same time H_2_O_2_ is generated outside the cell by the action of the cell-surface xanthine oxidoreductase on hypoxanthine, also produced among others by Bs29784.

## 5. Conclusions

In conclusion, this study identified hypoxanthine and nicotinic acid as two important metabolites produced by *B. subtilis* strain 29784. The probiotic was shown to be metabolically active, producing these two metabolites in the intestine of broilers. These metabolites contribute, at least in part, to the interaction of Bs29784 with both the host and the microbiome, either through direct anti-inflammatory or anti-bacterial properties or by increasing the abundance of beneficial butyrate-producing bacteria in the cecum, potentially through cross-feeding.

## Figures and Tables

**Table 1:** Metabolites that are significantly increased (fold change > 2 and *p* < 0.05) after 24 h growth of *B. subtilis* strain 29784 in LB medium.

Metabolite	Area Ratio (Mean ± SD)		Fold Change	*p*-Value
	Blank	Bs29784		
Hypoxanthine	0.173 ± 0.002	1.844 ± 0.086	106.40	<0.0001
Nicotinic acid	0.218 ± 0.030	1.853 ± 0.104	8.51	<0.0001
Ethanolamine	0.007 ± 0.003	0.061 ± 0.016	8.67	0.005
Uracil	0.241 ± 0.004	1.652 ± 0.392	6.85	0.003
Pantothenate	0.001 ± 0.001	0.022 ± 0.002	2.03	0.002
3-Hydroxypyridine	0.006 ± 0.003	0.014 ± 0.001	2.16	0.015
2.5-dimethylpyrazine	0.005 ± 0.000	0.012 ± 0.003	2.47	0.017
Thymine	0.014 ± 0.007	0.034 ± 0.004	2.51	0.011

**Table 2:** Taxonomic and functional alpha diversity of ileal and cecal microbial communities from broilers fed either a control or a Bs29784-supplemented feed.

	Control	Bs29784	*p*-Value
**ILEUM**			
Taxonomic alpha diversity			
nOTUs	98.8 ± 29.95	90 ± 16.02	0.69
Chao1	125.31 ± 49.39	107.59 ± 24.07	0.69
Shannon	1.72 ± 0.40	1.06 ± 0.43	0.032 *
Functional alpha diversity			
nKOs	4487 ± 257.13	4522.6 ± 145.87	1
Chao1	4656.89 ± 375.39	4743.67 ± 298.32	1
Shannon	7.40 ± 0.23	7.16 ± 0.18	0.15
**CECUM**			
Taxonomic alpha diversity			
nOTUs	142.8 ± 5.45	181.2 ± 25.08	0.056
Chao1	157.74 ± 7.13	196.50 ± 30.77	0.15
Shannon	2.91 ± 0.41	3.26 ± 0.58	0.42
Functional alpha diversity			
nKOs	4228.4 ± 111.10	4205.0 ± 76.41	1
Chao1	4554.97 ± 210.53	4414.80 ± 191.05	0.42
Shannon	7.71 ± 0.13	7.39 ± 0.14	0.016 *

* Significant differences between the control and Bs29784 group (*p* < 0.05).

**Table 3:** Differentially abundant genera in the ileal or cecal microbiota.

Phylum	Class	Family	Genus	Mean Abundance (%)		Log2 Fold Change	Adjusted *p*-Value
				Control	Bs29784		
ILEUM							
*Actinobacteria*	*Actinobacteria*	*Beutenbergiaceae*	Ambiguous taxa *Beutenbergiaceae*	0.046	0.000	−23.36	<0.001
*Firmicutes*	*Bacilli*	*Bacillaceae*	*Bacillus*	0.000	0.121	7.54	<0.001
*Firmicutes*	*Bacilli*	*Lactobacillaceae*	*Pediococcus*	0.250	0.035	−4.32	0.019
*Firmicutes*	*Bacilli*	*Leuconostocaceae*	*Weissella*	0.253	0.002	−7.20	<0.001
*Firmicutes*	*Clostridia*	*Peptostreptococcaceae*	Ambiguous taxa *Peptostreptococcaceae*	0.054	0.000	−22.66	<0.001
*Firmicutes*	*Negativicutes*	*Veillonellaceae*	Family *Veillonellaceae*	0.062	0.000	−22.91	<0.001
*Proteobacteria*	*Gammaproteobacteria*	*Enterobacteriaceae*	Ambiguous taxa *Enterobacteriaceae*	0.473	0.051	−3.71	0.007
*Proteobacteria*	*Gammaproteobacteria*	*Enterobacteriaceae*	*Enterobacter*	0.045	0.002	−6.32	0.001
*Proteobacteria*	*Gammaproteobacteria*	*Enterobacteriaceae*	*Klebsiella*	0.058	0.002	−6.09	0.007
CECUM							
*Firmicutes*	*Bacilli*	*Enterococcaceae*	*Enterococcus*	1.746	4.865	2.30	0.016
*Firmicutes*	*Clostridia*	*Clostridiales vadinBB60 group*	uncultured bacterium_Clostridiales vadinBB60 group	0.000	0.956	12.51	<0.001
*Firmicutes*	*Clostridia*	*Lachnospiraceae*	*[Eubacterium] hallii group*	0.000	0.074	22.48	<0.001
*Firmicutes*	*Clostridia*	*Lachnospiraceae*	GCA-900066575	0.000	0.062	22.47	<0.001
*Firmicutes*	*Clostridia*	*Lachnospiraceae*	Lachnospiraceae FCS020 group	0.004	0.219	7.32	<0.001
*Firmicutes*	*Clostridia*	*Lachnospiraceae*	Lachnospiraceae NK4A136 group	0.000	0.556	25.64	<0.001
*Firmicutes*	*Clostridia*	*Peptostreptococcaceae*	*Clostridioides*	0.000	0.066	23.25	<0.001
*Firmicutes*	*Clostridia*	*Ruminococcaceae*	*Negativibacillus*	0.000	0.693	11.10	<0.001
*Firmicutes*	*Clostridia*	*Ruminococcaceae*	*Ruminiclostridium 9*	0.239	1.359	2.93	0.0461
*Firmicutes*	*Clostridia*	*Ruminococcaceae*	Ruminococcaceae UCG-013	0.000	0.008	27.52	<0.001
*Firmicutes*	*Negativicutes*	*Veillonellaceae*	Family_*Veillonellaceae*	1.272	0.000	−27.55	<0.001
*Firmicutes*	*Negativicutes*	*Veillonellaceae*	*Sporomusa*	3.657	0.000	−28.07	<0.001
*Proteobacteria*	*Gammaproteobacteria*	*Enterobacteriaceae*	Ambiguous_taxa_Enterobacteriaceae	5.518	0.758	−2.48	<0.001
*Proteobacteria*	*Gammaproteobacteria*	*Enterobacteriaceae*	*Enterobacter*	0.718	0.059	−3.03	0.004
*Proteobacteria*	*Gammaproteobacteria*	*Enterobacteriaceae*	*Klebsiella*	3.221	0.745	−2.33	0.006

Significant differences in genus level abundance in the ileal or cecal microbiota from birds fed the Bs29784-supplemented diet as compared to the control diet. The taxonomic classification and the log2 fold change (log2FC) (Bs29784/control) of the DESeq2-normalized abundance of each genus are shown. Positive values indicate an increase in abundance of the respective genus in the Bs29784 group, while negative values indicate a decrease.

## Data Availability

The raw sequencing data are available on NCBI SRA under the BioProject ID PRJNA716565. All other data are available from the corresponding author on reasonable request.

## Supplementary Materials

The following are available online at https://www.mdpi.com/article/10.3390/ani11051335/s1, Table S1: Metabolites detected in either blank LB medium or after 24 h growth of *B. subtilis* strain Bs29784 on LB medium, Table S2: Differentially abundant OTUs in the ileal microbiome of birds fed either the control or Bs29784-supplemented diet, Table S3: Differentially abundant OTUs in the cecal microbiome of birds fed either the control or Bs29784-supplemented diet, Table S4: Differentially abundant gut metabolic modules (GMM) in the ileal microbiome of birds fed either the control or Bs29784-supplemented diet, Table S5: Differentially abundant gut metabolic modules (GMM) in the cecal microbiome of birds fed either the control or Bs29784-supplemented diet, Table S6: Mean and SEM of the number of times a bacterial family contributes to a specific module in the ileum, Table S7: Mean and SEM of the number of times a bacterial family contributes to a specific module in the cecum.
